# Single-incision retroperitoneal laparoscopic repair of superior lumbar hernia using self-fixating ProGrip mesh: A case report

**DOI:** 10.1016/j.ijscr.2020.01.034

**Published:** 2020-02-06

**Authors:** Yujiro Nakahara, Masaki Wakasugi, Satoshi Nagaoka, Satoshi Oshima

**Affiliations:** a Department of Gastroenterological Surgery, Osaka Police Hospital, 10-31 Kitayama-cho, Tennoji-ku, Osaka, 543-0035, Japan; b Department of Surgery, Kinki Central Hospital, 3-1 Kurumaduka, Itami, Hyogo 664-8533, Japan; c Department of Surgery, Osaka Rosai Hospital, 1179-3 Nagasone-cho, Kita-ku, Sakai, Osaka, 591-8025, Japan

**Keywords:** Superior lumbar hernia, Single-incision retroperitoneal laparoscopic repair, Laparoscopic self-fixating mesh

## Abstract

**Introduction:**

Lumbar hernia is rare and represents less than 1–2% of all abdominal hernias. There are mainly two types of lumbar hernia: superior lumbar hernia and inferior lumbar hernia.

**Case presentation:**

A 65-year-old woman was admitted complaining of a mass in her left lumbar area. Under a diagnosis of superior lumbar hernia, single-incision laparoscopic retroperitoneal repair was performed. A single, 2-cm-long incision was made and the retroperitoneal space was dissected gradually. The hernia orifice was recognized and hernia sac was slipped from the hernia orifice. The collateral branch of subcostal nerve and iliohypogastric nerve were recognized. Laparoscopic self-fixating mesh was placed to cover the hernia orifice without mesh fixation. The patient remained well with no signs of recurrence.

**Discussion:**

In laparoscopic lumbar hernia repair, it is important to be careful not to damage subcostal nerve and iliohypogastric nerve. Self-fixating mesh without fixation is useful due to the prevention from nerve injury. To our best knowledge, this is the first report of single-incision laparoscopic repair for superior lumbar hernia. Single-incision laparoscopic surgery could provide good cosmetic results with minimal incision.

**Conclusion:**

We successfully performed single-incision retroperitoneal laparoscopic repair of superior lumbar hernia using self-fixating mesh.

## Highlights

• Superior lumbar hernia is an uncommon hernia.
• We performed single-incision retroperitoneal laparoscopic repair.
• Self-fixating mesh without fixation is useful because of no risk of nerve injury.

## 1 Introduction

Lumbar hernia is an uncommon hernia. Only about 300 cases of lumbar hernia have been reported in the literature [1]. Lumbar hernia generally protrudes through two anatomical areas: the superior lumbar triangle of Grynfelt-Lesshaft and the inferior lumbar triangle of Petit. Hernia repair using a surgical mesh is often performed for lumbar hernia. There are three typical surgical approaches: the anterior approach, laparoscopic transabdominal approach and retroperitoneal laparoscopic approach. We here present a case of superior lumbar hernia that was successfully repaired by single-incision retroperitoneal laparoscopic approach using self-fixating mesh without mesh fixation. This work has been reported in line with the SCARE criteria [2].

## 2 Case presentation

### 2.1 Patient

**Fig. 1:**
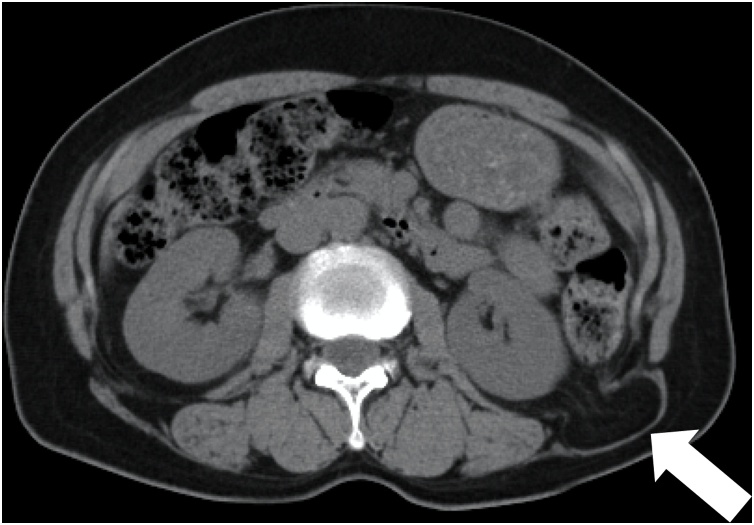
Computed tomography revealed the left superior lumbar hernia (white arrow). The size of hernia orifice was 20 × 14 mm.

A 65-year-old woman was admitted to our hospital with a complaint of a mass in her left lumbar area. She had a previous history of appendectomy at the age of 19 and had diabetes, hypertension, angina and hyperlipidemia as comorbidities. On physical examination, reducible soft mass was located in the left lumbar area. Computed tomography showed a defect of transverse fascia in the left lumbar area with the perirenal fat protruding through the defect. The size of hernia orifice was 20 × 14 mm (Fig. 1). Under a diagnosis of superior lumbar hernia, the patient underwent single-incision retroperitoneal laparoscopic repair.

### 2.2 Surgical technique

**Fig. 2:**
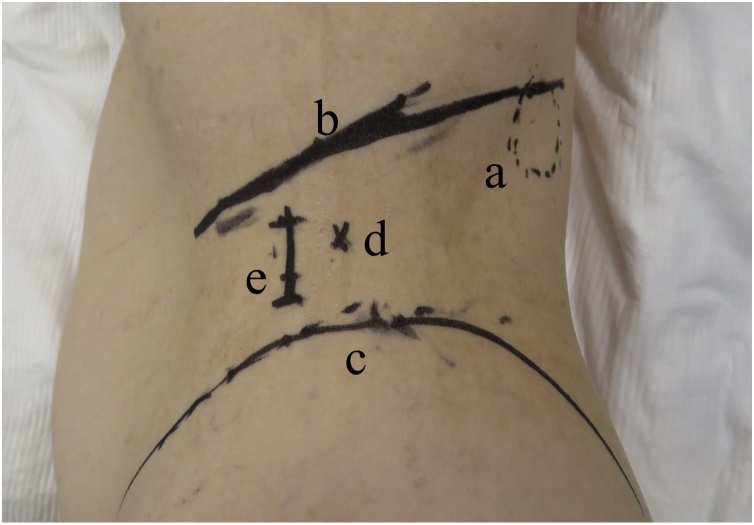
Patient positioned in the right lateral decubitus. Incision was made 1 cm ventral from the middle point between the 12th rib and superior anterior iliac spine on the middle axillary line. a: Superior lumbar hernia. b: The 12th rib line. c: Superior anterior iliac spine. d: The middle point between the 12th rib and superior anterior iliac spine on the middle axillary line. e: Incision line.

**Fig. 3:**
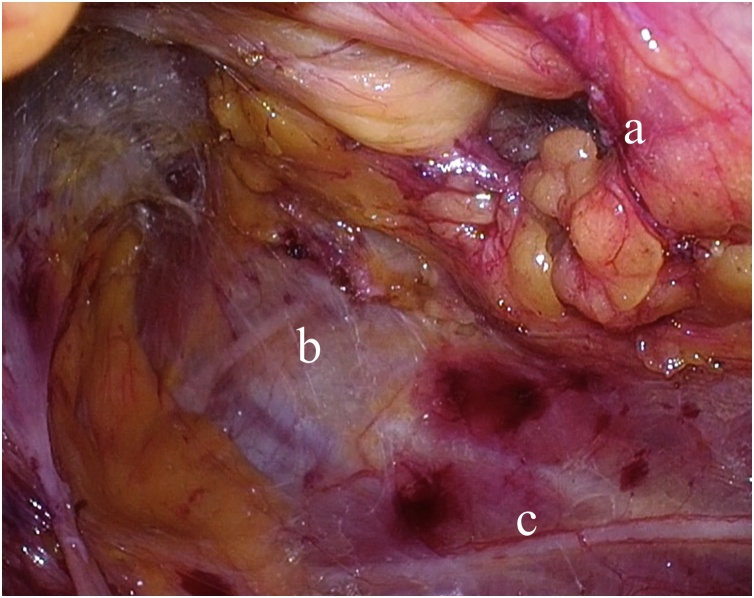
The retroperitoneal space was dissected. The hernia orifice, the collateral branch of subcostal nerve and iliohypogastric nerve were recognized. a: The hernia orifice. b: The collateral branch of subcostal nerve. c: Iliohypogastric nerve.

**Fig. 4:**
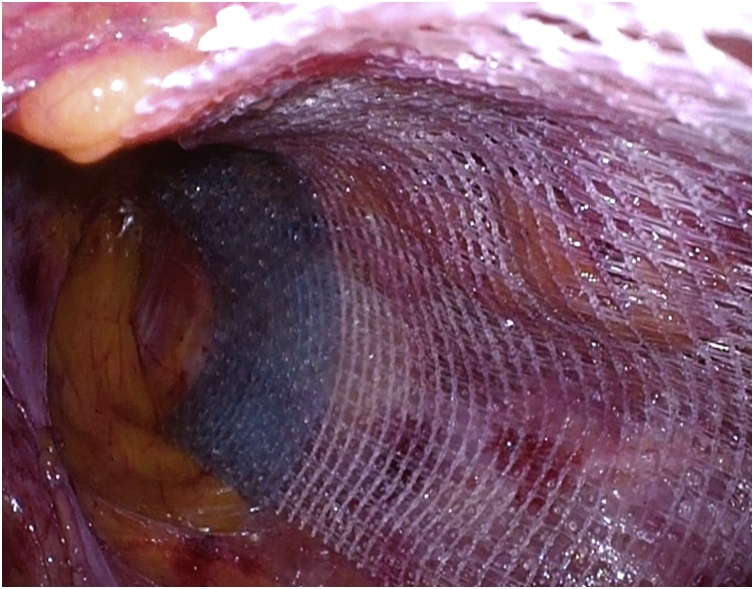
ProGrip™ laparoscopic self-fixating mesh was placed to cover the hernia orifice without mesh fixation.

Under general anesthesia, the patient was placed in the right lateral decubitus position. A single, 2-cm-long incision was made 1 cm ventral from the middle point between the 12th rib and superior anterior iliac spine on the middle axillary line (Fig. 2). External oblique muscle, internal oblique muscle and transverse abdominal muscle were each split to reach retroperitoneal space. After placing a Lap-Protector Mini (Hakko Co., Nagano, Japan) in the retroperitoneal space, three 5-mm trocars (one for a 5-mm flexible scope and two for surgical devices) were inserted through a single-port access device (EZ Access; Hakko). The EZ access with three trocars was attached to the Lap-Protector Mini to maintain the inflation of the preperitoneal space with carbon dioxide (CO_2_) gas, and the retroperitoneal space was gradually dissected, using laparoscopic instruments without a dissection balloon. The hernia orifice was recognized and hernia sac was slipped from the hernia orifice. The collateral branch of subcostal nerve and iliohypogastric nerve were recognized (Fig. 3). ProGrip™ laparoscopic self-fixating mesh of 15 × 10 cm (Medtronic, Dublin, Ireland) was placed to cover the hernia orifice without mesh fixation (Fig. 4). The retroperitoneal space was carefully deflated to avoid displacing the mesh. The sheaths of transverse abdominal muscle and external oblique muscle were each closed with 2–0 absorbable suture, and the skin was closed with 4–0 absorbable suture.

The operative time was 50 min, and the bleeding volume was minimal. The postoperative course was uneventful. Food intake and normal activities restarted the next day after surgery, and she was discharged five days after surgery. At the 9-month follow-up, she remained well, with no signs of recurrence.

## 3 Discussion

Lumbar hernia is rare and represents less than 1–2% of all abdominal hernias [3]. There are mainly two types of lumbar hernia: superior lumbar hernia and inferior lumbar hernia. Superior lumbar hernia generally protrudes through the superior lumbar triangle of Grynfelt-Lesshaft. The superior lumbar tringle is formed medially by the sacrospinalis, laterally by the posterior border of the internal oblique muscle, and superiorly by the 12th rib and the serratus posterior inferior muscles. The floor of the superior lumbar triangle is the transversalis fascia and its roof is the external abdominal oblique muscle [4]. The superior triangle is an anatomically weak area.

There are no standard surgical methods of superior lumbar hernia due to the relative rarity of this disease and lack of collective experience. Direct suturing of hernia orifice and reinforcement using muscle flaps have been performed, however, early recurrence cases have been reported [5]. It is suggested that the genesis of inguinal hernia was associated with muscle degeneration and weakening, damage to the vascular structures and nerve degeneration [[6], [7], [8]]. Therefore, tension-free hernia repair using a surgical mesh is often performed in recent years. There are three typical surgical approaches: the anterior approach, laparoscopic transabdominal approach and retroperitoneal laparoscopic approach.

Laparoscopic repair of lumbar hernia was first reported by Heniford et al. in 1997 [9]. A prospective study comparing open and laparoscopic lumbar hernia repair suggested that laparoscopic approach was safe, effective, and more efficient than open repair [10]. It is also described that laparoscopic pre-peritoneal approach is preferable in small, simple primary lumbar hernia [11]. Therefore, we selected retroperitoneal laparoscopic approach in this case. We used ProGrip™ laparoscopic self-fixating mesh of 15 × 10 cm because we considered that the mesh size of 15 × 10 cm was appropriate to cover the hernia orifice in this case.

However, laparoscopic repair of superior lumbar hernia has a risk of nerve injury [12]. The collateral branch of subcostal nerve and iliohypogastric nerve run in this area. It is very important to be careful not to damage both nerves in laparoscopic lumbar hernia repair. We recognized both nerves during surgery and used ProGrip™ laparoscopic self-fixating mesh without mesh fixation. There has been only one report of laparoscopic lumbar hernia repair using self-fixating mesh without traumatic fixation [13]. We suggest that laparoscopic lumbar hernia repair using self-fixating mesh without mesh fixation is useful due to the prevention from nerve injury.

We performed single-incision retroperitoneal laparoscopic repair. To the best of our knowledge, this is the first report of single-incision laparoscopic repair for superior lumbar hernia. We had performed more than 300 single-incision laparoscopic procedures for totally extraperitoneal repair (SILS-TEP) for adult inguinal hernia from May 2016 to December 2018, and we are familiar with the procedure of SILS-TEP [14]. Therefore, the technique of single-incision retroperitoneal laparoscopic repair was feasible for us. Retroperitoneal laparoscopic approach might be safe because of low risk to damage abdominal organs without accessing the abdominal cavity [13]. In addition, single-incision laparoscopic surgery, with minimal incision, provides good cosmetic results [15].

## 4 Conclusion

We successfully repaired superior lumbar hernia by single-incision retroperitoneal laparoscopic approach using self-fixating mesh without mesh fixation. Single-incision retroperitoneal laparoscopic repair of superior lumbar hernia using self-fixating mesh is considered to be a useful method because of good cosmetic results and no risk to damage nerves by mesh fixation.

## Conflicts of interest

The authors declare no conflict of interest.

## Funding

The authors received no specific funding for this work.

## Ethical approval

This case report is exempt from ethical approval in our institution.

## Consent

Written informed consent was obtained from the patient, and patient anonymity was preserved.

## Author contribution

Yujiro Nakahara: study concept, writing the paper

Masaki Wakasugi: study concept, writing the paper

Satoshi Nagaoka: review and revision of the paper

Satoshi Oshima: review and revision of the paper

## Registration of research studies

This manuscript is a case report not a research.

## Guarantor

Satoshi Oshima.

## Provenance and peer review

Not commissioned, externally peer-reviewed.
